# Supplementary material for: Repeatedly adopting power postures does not affect hormonal correlates of dominance and affiliative behavior
Source: PeerJ. 2019 Jun 17;7:e6726. doi: 10.7717/peerj.6726 (PMC6585898; doi:10.7717/peerj.6726)
Supplement: Supplemental Information 1 — Posture, Time and Posture*Time effects in ANOVAs conducted on the full sample before exclusion of outliers more than three absolute deviations above the median. η2p: partial eta-squared, η2G: generalized eta-squared. [file peerj-07-6726-s001.docx]

|  | **Effect** | **df n** | **df d** | **F** | **p** | **η^2^*_p_*** | **η^2^*_G_*** |
| --- | --- | --- | --- | --- | --- | --- | --- |
| Cortisol | Posture | 1 | 80 | 1,17 | 0,283 | 0,01 | 0,01 |
|  | Time | 2 | 160 | 49,73 | 0,000 | 0,38 | 0,11 |
|  | Posture*Time | 2 | 160 | 0,57 | 0,567 | 0,01 | 0,00 |
| Testosterone | Posture | 1 | 80 | 0,31 | 0,582 | 0,00 | 0,00 |
|  | Time | 2 | 160 | 16,60 | 0,000 | 0,17 | 0,02 |
|  | Posture*Time | 2 | 160 | 1,14 | 0,322 | 0,01 | 0,00 |
| Progesterone | Posture | 1 | 80 | 1,56 | 0,215 | 0,02 | 0,02 |
|  | Time | 2 | 160 | 32,17 | 0,000 | 0,29 | 0,04 |
|  | Posture*Time | 2 | 160 | 0,35 | 0,703 | 0,00 | 0,00 |
